# Use of point-of-care ultrasound in a low-resource setting to diagnose Achilles tendon rupture and avulsion fracture of the calcaneal bone

**DOI:** 10.1186/s12245-023-00544-7

**Published:** 2023-10-03

**Authors:** Naheed Habibullah, Jamil Dayo, Salman Muhammad Soomar, Noman Ali

**Affiliations:** Department of Emergency Medicine, Aga Khan University, Karachi, 74800 Pakistan

**Keywords:** Point-of-care, Ultrasound, Achilles tendon rupture, Emergency medicine, Calcaneal avulsion

## Abstract

**Background:**

Point-of-care ultrasound (POCUS) is becoming more prevalent in recent years for evaluating patients presenting with musculoskeletal injuries in the emergency department (ED). This imaging modality has been utilized to diagnose soft tissue and bony injuries accurately, obtain appropriate consultation, and perform timely interventional procedures in the ED.

**Case presentation:**

We present the case of a 55-year-old man who presented to the ED with significant left ankle pain following a ground-level fall. His physical examination showed swelling and tenderness around the ankle. POCUS examination aided the rapid and accurate detection of acute Achilles tendon rupture.

**Conclusion:**

This case demonstrates that POCUS is a valuable diagnostic tool in evaluating patients with a suspected Achilles tendon rupture, especially in a resource-limited setting.

## Background

Point-of-care ultrasound (POCUS) is becoming more prevalent in recent years for evaluating patients presenting with musculoskeletal injuries in the emergency department (ED). POCUS has been utilized to diagnose soft tissue and bony injuries accurately, obtain appropriate consultation, and perform timely interventional procedures in ED [1, 2]. Recent research studies have shown that POCUS can accurately identify long bone and metatarsal fractures, ligament injuries, and joint effusions distinguishing cellulitis from abscesses [3–6]. History and physical examination alone can miss up to 20% of tendon injuries in the initial phase, resulting in difficult repair and poor functional strength [5]. Because most tendons are relatively superficial, POCUS can easily identify these injuries. According to the literature, emergency physicians can use POCUS to identify tendon injuries with 100% sensitivity and 95% specificity [7]. We present a case of a partial Achilles tendon rupture in a middle-aged male patient who was rapidly diagnosed by POCUS in the ED, resulting in early management and orthopedic outpatient referral.

## Case presentation

**Fig. 1:**
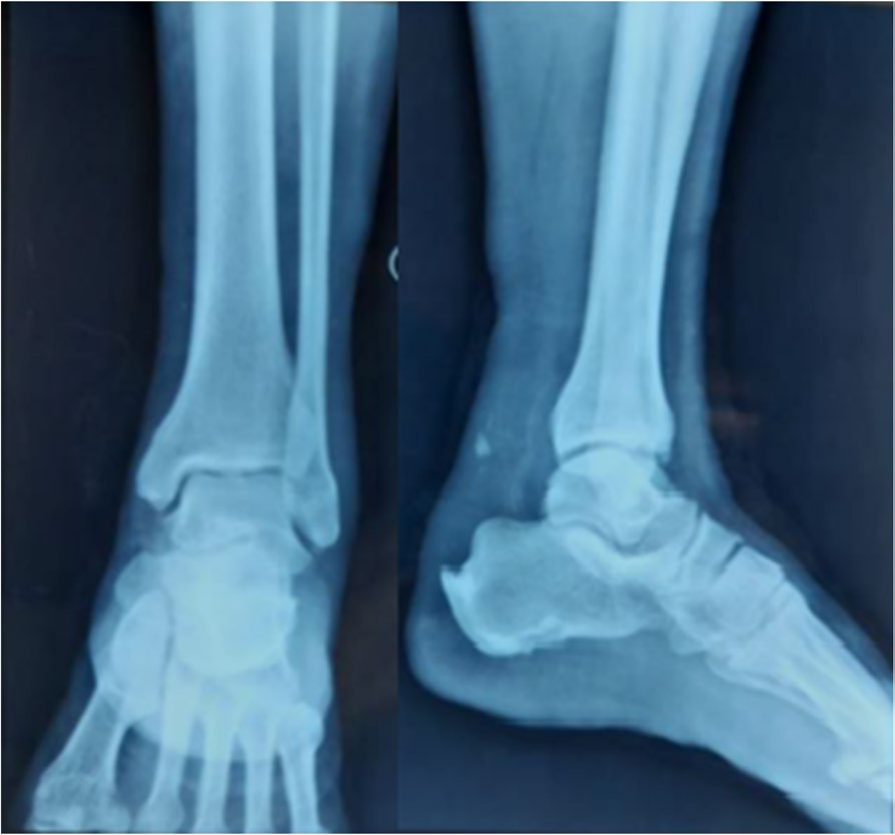
X-ray of the left foot showing an avulsion fracture of the calcaneal bone

A 55-year-old man with a history of diabetes mellitus and seronegative rheumatoid arthritis presented to the ED of the Aga Khan University Hospital (AKUH), Karachi, with a complaint of pain and swelling in the left ankle and inability to bear weight on his left lower limb after a history of ground-level fall 2 h back. He initially went to an outside hospital, where an X-ray of his foot showed an avulsion fracture of the calcaneal bone (Fig. 1).

**Fig. 2:**
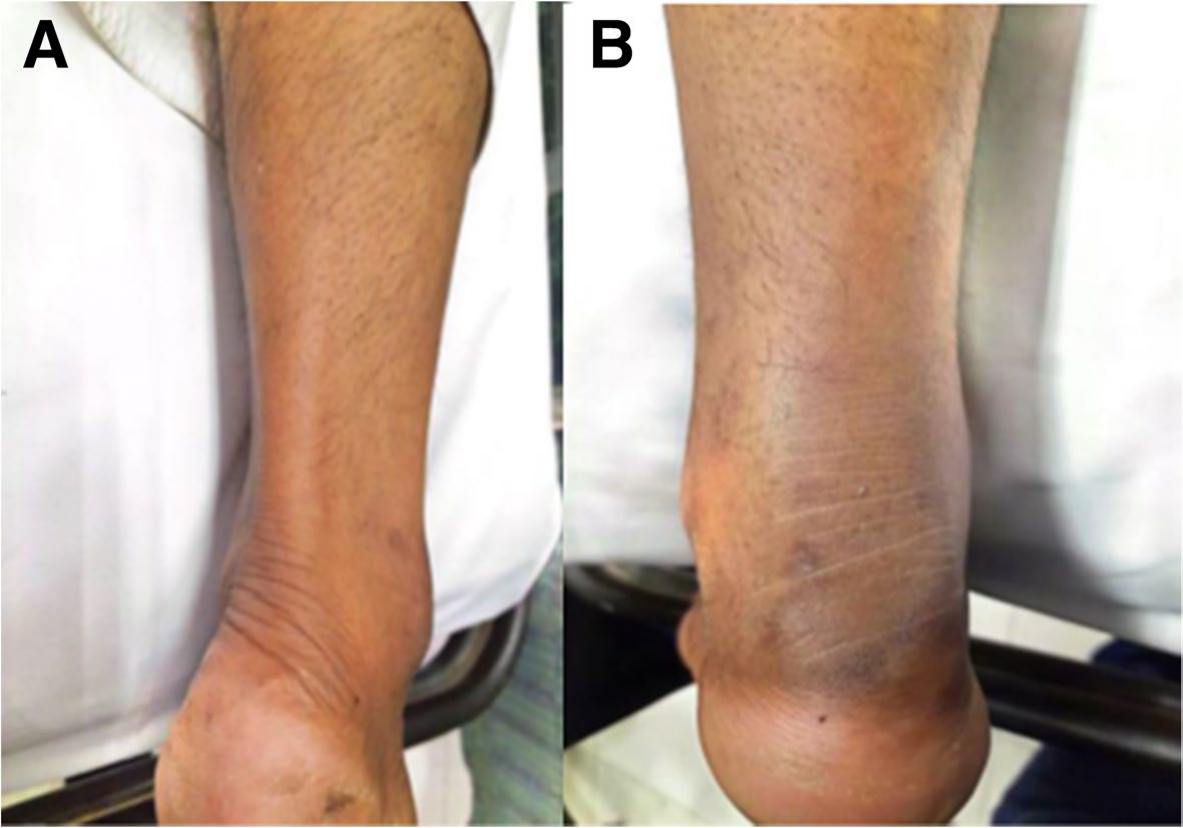
**a** Loss of normal resting tone and posture of the left foot with **b** loss of tendon definition

He was referred to AKUH for an orthopedic opinion. On arrival, he was vitally stable. The general physical and systemic examination was unremarkable. His musculoskeletal examination showed significant swelling and tenderness around the left ankle and difficulty bending the foot downward (plantarflexion) (Fig. 2). He had no sensory deficits and no overlying skin changes or open wounds.

**Fig. 3:**
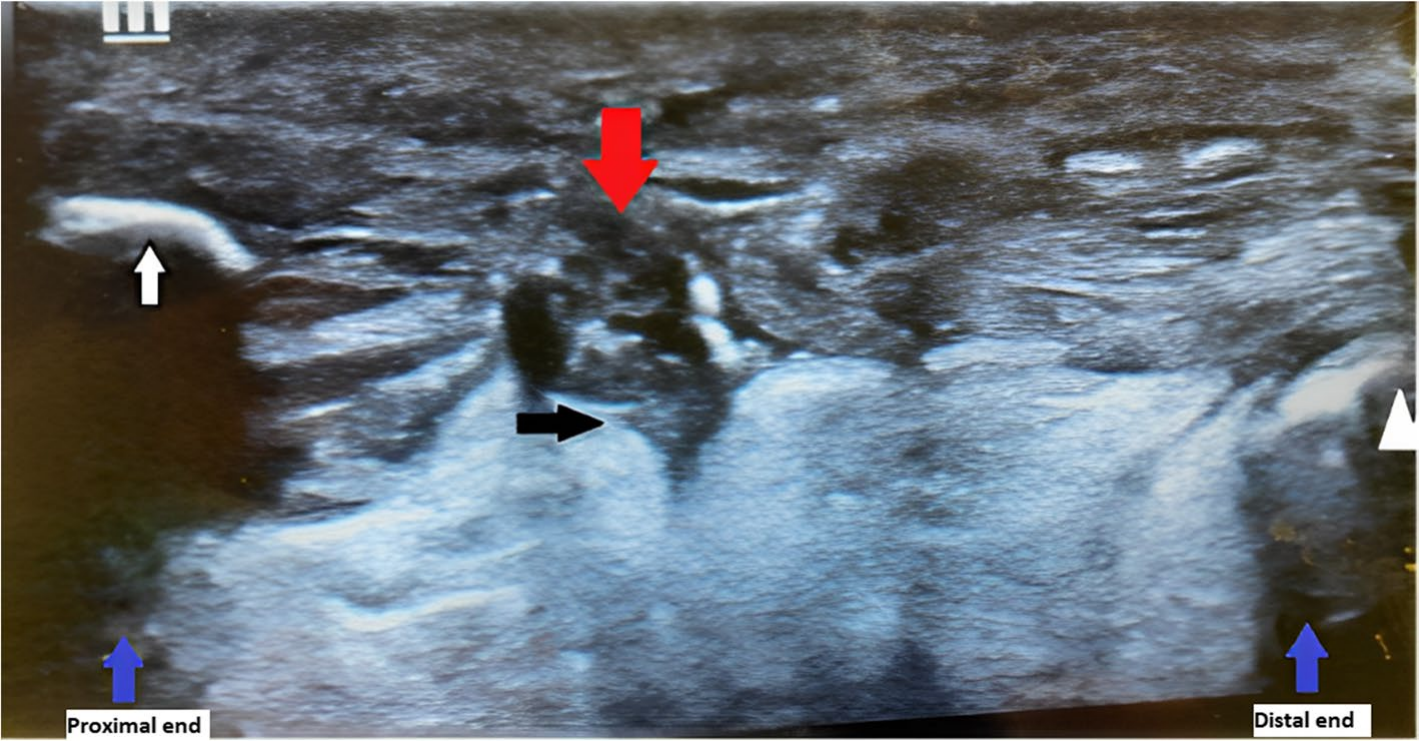
Point-of-care ultrasound with a longitudinal view of the Achilles tendon. The white arrow indicates an avulsed fragment of calcaneum, the black arrow indicates the torn edge of the Achilles tendon, and the white triangle indicates calcaneum. Blue arrows indicate the proximal end and distal end (left to right). The red arrow indicates a possible hematoma between the torn edges of the Achilles tendon

The on-call ED consultant performed a POCUS. The patient was lying prone with his feet hanging off the bed. A high-frequency linear probe was selected and placed on the posterior ankle in the long axis in a neutral position, which showed the disruption of the typical linear tendon fibers. Dynamic testing revealed the movement of the distal stump of the Achilles tendon adjacent to hypoechoic fluid, most likely reactive edema or blood from the acute rupture. Hyperechogenicity was also noted at the proximal end of the Achilles tendon, with acoustic shadowing probably representing an avulsed fragment of the calcaneum (Fig. 3).

His foot was splinted in the plantar flexion position, and an orthopedic follow-up was arranged in two days. His MRI (magnetic resonance imaging) of the foot was done on an outpatient basis as advised by the orthopedic consultant, who was reported as an “avulsion fracture of the calcaneum at the attachment site of the Achilles tendon with retraction of the partially ruptured Achilles tendon. The retracted ruptured end of the Achilles tendon is at the level of the distal articular margin of the tibia and is approximately 47mm from its attachment site. Significant surrounding soft tissue edema and hematoma are noted.” He was treated conservatively with analgesics and non-weight-bearing status.

## Discussion

An Achilles tendon rupture diagnosis in the ED is often made solely based on history and physical exam; however, up to 20% of ruptures go unnoticed during the hyperacute phase and are frequently misdiagnosed as an ankle sprain [8]. Clinical examinations for diagnosing Achilles tendon rupture using diagnostic maneuvers, including the Thompson test and the presence of a palpable tendon defect, have shown sensitivities between 73 and 96%. Misdiagnosis and delayed treatment can lead to chronic discomfort, nonunion, and mobility issues [9]. MRI is often used to assess musculoskeletal complaints and is the study of choice when tendon rupture is suspected. This imaging modality provides greater anatomic detail and accuracy in detecting partial Achilles tendon rupture [10]. However, the utility of MRI in the ED is limited due to the longer scan times and higher expenses.

POCUS is a useful ancillary tool for evaluating soft tissue injuries in real time and at a lower cost. The sensitivity of ultrasound for detecting this type of injury ranges from 79 to 100%. The clinical utility of ultrasound in Achilles tendon injuries has previously been demonstrated [11–14]. It enables rapid bedside confirmation and distinguishes between partial and complete tears [15]. The patient should lie prone with feet hanging off the bed. A high-frequency linear probe should be used as it provides better resolution for superficial structures. The probe should be placed and slide gently over the Achilles tendon to identify different views. In a ruptured Achilles tendon, there will be a discontinuity in the tendon fibers, with the surrounding anechoic area representing the blood or edema. Dynamic assessment may show a lack of movement in the torn area during foot extension and flexion and can differentiate between full or partial-thickness tendon rupture.

Our case demonstrates the importance of POCUS in early decision-making and management of patients in a resource-limited country. Literature has shown that the POCUS has a positive impact on clinical decision-making in resource-limited settings, resulting in a decreased length of stay in EDs and a measurable reduction in planned referrals [16, 17]. Moreover, using POCUS also reduces the safety issues and costs associated with invasive and conventional testing like CT-scan and MRI [18].

## Conclusion

POCUS is a practical and valuable modality to diagnose Achilles tendon rupture in patients presenting to the ED with a history of an ankle injury at the bedside. In combination with clinical history and examination, this can reduce ED stay and the number of expensive and time-consuming imaging resulting in cost-effectiveness, early decision-making, and planned referrals. POCUS is especially well-suited for resource-limited settings due to its quick accessibility of results and independence from additional infrastructure and staff (radiologists). As advancements continue, affordability and portability will further ease the adoption of POCUS over time.

## Data Availability

Available on reasonable request to noman.ali@aku.edu.

## Abbreviations

AKUH: Aga Khan University Hospital
ED: Emergency department
MRI: Magnetic resonance imaging
POCUS: Point-of-care ultrasound
